# Genomic RNA sequence of feline coronavirus strain FCoV C1Je

**DOI:** 10.1016/j.jfms.2006.12.002

**Published:** 2007-06

**Authors:** Charlotte Dye, Stuart G. Siddell

**Affiliations:** Department of Cellular and Molecular Medicine, Medical and Veterinary Sciences, University of Bristol, Bristol BS8 1TD, United Kingdom

## Abstract

This paper reports the first genomic RNA sequence of a field strain feline coronavirus (FCoV). Viral RNA was isolated at post mortem from the jejunum and liver of a cat with feline infectious peritonitis (FIP). A consensus sequence of the jejunum-derived genomic RNA (FCoV C1Je) was determined from overlapping cDNA fragments produced by reverse transcriptase polymerase chain reaction (RT-PCR) amplification. RT-PCR products were sequenced by a reiterative sequencing strategy and the genomic RNA termini were determined using a rapid amplification of cDNA ends PCR strategy. The FCoV C1Je genome was found to be 29,255 nucleotides in length, excluding the poly(A) tail. Comparison of the FCoV C1Je genomic RNA sequence with that of the laboratory strain FCoV FIP virus (FIPV) 79-1146 showed that both viruses have a similar genome organisation and predictions made for the open reading frames and *cis*-acting elements of the FIPV 79-1146 genome hold true for FCoV C1Je. In addition, the sequence of the 3′-proximal third of the liver derived genomic RNA (FCoV C1Li), which encompasses the structural and accessory protein genes of the virus, was also determined. Comparisons of the enteric (jejunum) and non-enteric (liver) derived viral RNA sequences revealed 100% nucleotide identity, a finding that questions the well accepted ‘internal mutation theory’ of FIPV pathogenicity.

Feline coronavirus (FCoV) infection is extremely common in cats worldwide. In the United Kingdom approximately 40% of the domestic cat population is seropositive and where cats are housed together in multi-cat households, this figure increases still further to around 90% (Addie and Jarrett, 1992, Sparkes et al, 1992, Addie, 2000). Natural infections with FCoV are usually transient, although a significant percentage of infections may become persistent (Addie and Jarrett 2001). Most infections are asymptomatic or result in mild, self-limiting gastrointestinal disease and in these cases, the causative agent is known as feline enteric coronavirus (FECV). In a small percentage of animals (<5%), however, a fatal multi-systemic, immune-mediated disease occurs and this is known as feline infectious peritonitis (FIP) (Pedersen 1995). The virus associated with FIP is referred to as FIP virus (FIPV) and it is proposed that cats acquire FIPV by mutation of an endogenous FECV (Poland et al, 1996, Vennema et al, 1998). This hypothesis is known as the ‘internal mutation theory’ and is widely accepted. Despite this, any genetic differences between FECV and FIPV that can account for their different pathogenicity remain to be identified.

There are two types of FCoV that can be distinguished by serology and by sequence analysis. Type I viruses are most prevalent in the field and account for approximately 80% of all infections (Hohdatsu et al, 1992, Addie et al, 2003). Type II viruses are less prevalent and are characterised by recombination events that result in the replacement of the FCoV spike glycoprotein gene with the equivalent gene of canine enteric coronavirus (CCoV) (Herrewegh et al 1998). There is no evidence that either type is more commonly associated with FIP in natural infections (Benetka et al 2004). The majority of research on FCoV to date has concentrated on the investigation of type II strains, most notably FIPV 79-1146, because they replicate well in cell culture and are, therefore, easy to work with in the laboratory. However, FIPV 79-1146 is unlikely to be representative of coronavirus strains that are currently circulating within domestic cat populations. Firstly, it was isolated in America in 1979 and the geographical and time differences make it unlikely that this strain would be in current circulation in the UK. Secondly, it is a type II strain, which represent only 10–20% of field isolates (Hohdatsu et al, 1992, Benetka et al, 2004, Kummrow et al, 2005). Thirdly, and most importantly, FIPV 79-1146 has been extensively passaged in cell culture, which increases the likelihood of mutation. We, therefore, felt it important that more clinically relevant strains should be investigated and genomic sequencing of a field strain virus obtained directly from clinical material was the first step in this process.

The ‘internal mutation theory’ states that FIP occurs when a cat is exposed to variants of FCoV that have mutated within the host and are able to disseminate from the gut (primary site of infection) by gaining the ability to replicate efficiently within macrophages (Pedersen, 1995, Poland et al, 1996, Vennema et al, 1998). This hypothesis has had many proponents and numerous speculations regarding the location of mutation(s) that could result in the alteration of pathogenicity have been made (Haijema et al, 2004, Rottier et al, 2005). It should, however, be noted that the difference between FCoV infection with and without FIP disease is believed to be quantitative rather than absolute (Meli et al, 2004, Rottier et al, 2005, Kipar et al, 2006). Most authors have concurred that although low-level monocyte-associated viraemia is found with FECV infection, this virus is mainly confined to the gut. This is in contrast to the highly pathogenic FIPV, which disseminates systemically with high viral titres. Thus, obtaining sequence data from enteric and non-enteric FCoVs found within individual cats with FIP may shed more light on any genetic differences between FECV and FIPV.

This paper presents the first genomic RNA sequence of a field strain FCoV. Viral RNA (designated FCoV C1Je) was isolated at post mortem from the jejunum of a cat with a histopathologically confirmed diagnosis of FIP. The sequence was analysed to identify *cis*-acting elements involved in the replication, transcription and translation of viral RNA and to identify the structural, non-structural and accessory proteins encoded by the genomic RNA. A direct comparison has been made with similar elements in the previously published genomic RNA of the laboratory strain FCoV, FIPV 79-1146. Furthermore, the structural and accessory gene regions of viral RNA isolated from the liver of the same cat (FCoV C1Li) were sequenced and the data derived from the enteric (jejunum) and non-enteric (liver) sources were compared.

## Methods

### Isolation of viral RNA

A 0.5 cm^3^ biopsy of jejunum and liver tissue from a cat with a diagnosis of FIP (confirmed by histopathology) was placed into 2 ml of ‘RNA later’ solution (Ambion, UK) at post mortem. This was stored at 4°C overnight. The ‘RNA later’ solution was then discarded and the tissue stored at −80°C. RNA was extracted from the tissue biopsies using a Machery–Nagel Nucleospin RNA II kit (ABgene, UK) according to the manufacturer's instructions. Briefly, tissue biopsies (30 mg) were added to 500 μl of lysis buffer with 1% (v/v) 2-mercaptoethanol in a 2-ml tube containing a stainless steel ball bearing. Samples were disrupted using a tissue lyser (Qiagen, UK) at a frequency of 30 revolutions per second for 2–4 min. Using a ‘shredder column’, 350 μl of the tissue lysate was homogenised and one volume of 70% ethanol was added before loading onto an ‘extraction column’. The sample was incubated with DNAse solution, washed three times, eluted in 2× 60 μl of RNAse-free water and stored at −80°C.

### cDNA synthesis and PCR amplification of viral sequences

**Fig 1:**
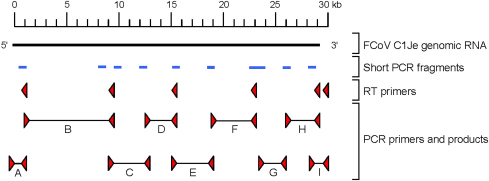
The positions of the RT and PCR primers used for the amplification of FCoV C1Je are shown in relation to the genomic RNA. The FCoV C1Je genomic RNA is illustrated in black (). Short C1Je PCR fragments amplified using primers specific for FIPV 79-1146 are shown in blue (). The sequence data derived using these initial primers were used to design C1Je specific primers for amplification of longer fragments. C1Je specific primers used for reverse transcription and PCR are highlighted in red (). PCR products are represented by a thin black line () joining the forward and reverse PCR primers and are labelled alphabetically from A to I.

Sequence data previously generated for the laboratory strain FCoV, FIPV 79-1146, were used to design primers for conventional reverse transcriptase polymerase chain reaction (RT-PCR) amplification of short lengths (100–500 bases) of the field strain RNA. These primers were chosen in regions that were expected to show sequence conservation on the basis of comparative analysis of published sequences. Subsequently, sequence data derived from these short PCR fragments were used to design field strain specific PCR primers that were used to amplify longer overlapping fragments spanning the entire genome (Fig 1).

For small RT-PCR fragments (<3 kb), Superscript II RNAse H^−^ RT (Invitrogen, UK) was used to reverse transcribe viral RNA. RNA and 5 pmol of reverse primer were incubated at 65°C for 5 min and then chilled on ice. The RNA/primer mix was then added to a 20 μl reaction containing 15 units of human placental ribonuclease inhibitor (HPRI), 1 mM deoxyribonucleotide equimolar mix of dATP, dCTP, dGTP and dTTP (dNTP), 0.01 M Dithiothreitol (DTT), 200 units of RT and 1× first strand buffer. The reaction was incubated at 42°C for 50 min followed by 94°C for 2 min. Samples were immediately chilled on ice and stored at −20°C. PCR amplification was undertaken using recombinant *Taq* DNA polymerase (Invitrogen, UK) in a 100 μl reaction containing 2 μl cDNA, 0.25 μM forward primer, 0.25 μM reverse primer, 0.8 mM dNTP, 5 units of DNA polymerase, 1× PCR buffer and 2 mM MgCl_2_. Reactions were incubated at 94°C for 2 min and then amplified using 35 cycles of 94°C for 20 s, 50–65°C for 20 s and 68°C for 1 min per kb of PCR product. Following a final incubation of 68°C for 10 min, the DNA was stored at 4°C.

For larger RT-PCR fragments (>3 kb), a one-step RT-PCR amplification was undertaken using a one-step PCR kit for long templates (Invitrogen, UK) according to the manufacturer's instructions. Briefly, a 50 μl reaction containing RNA, 15 pmol forward primer, 15 pmol reverse primer, 1 μl Superscript II RT/Platinum Taq HiFi DNA polymerase enzyme mix and 1× reaction buffer was incubated at 50°C for 30 min and 94°C for 2 min, followed by 35 cycles of 94°C for 15 s, 50–65°C for 30 s and 68°C for 1 min per kb of PCR product. The reaction was held at 72°C for 7 min and then stored at 4°C.

PCR products were purified using SigmaSpin post-reaction purification columns (Sigma–Aldrich, UK) or, if non-specific products were present, then gel purification was undertaken using a Qiagen gel purification kit (Qiagen, UK) according to the manufacturer's instructions. An aliquot of each purified DNA was electrophoresed in 1× TBE buffer at 100 V for 90 min on a 1% agarose/1× TBE gel containing 50 μl/ml ethidium bromide. DNA stocks were diluted to a final concentration of 100–300 ng/μl and stored at −20°C.

### 5′ RACE and 3′ RACE

3′- and 5′ Rapid amplification of cDNA ends (RACE) strategies were employed for amplification of the viral RNA termini. 3′ RACE was undertaken by reverse transcribing the RNA using primer 3′-RT followed by PCR amplification with primers F001 and P036 (Table 1). 5′ RACE was undertaken using a Generacer 5′ RACE kit (Invitrogen, UK) according to the manufacturer's instructions. Briefly, RNA was dephosphorylated using calf intestinal phosphatase, decapped using tobacco acid pyrophosphatase and ligated to a generacer RNA oligo using T4 RNA ligase. Phenol:chloroform extraction and ethanol precipitation of the RNA were undertaken between each step and the RNA was re-suspended in RNAse-free water. Reverse transcription was undertaken with Superscript II RNAse H^−^ RT (Invitrogen, UK) using primer F036 and PCR amplification was done with *Taq* DNA polymerase (Invitrogen, UK) using primers P037 and F036 (Table 1).

**Table 1:** RT and PCR primers used for the amplification of FCoV C1Je and FCoV C1Li RNA

Primer name	Use	Nucleotide sequence	Position on FCoV C1Je genome
P037	Forward PCR primer for fragment A	CGACTGGAGCACGAGGACACTGACAT	Designed on generacer RNA oligo
F036	RT and reverse PCR primer for fragment A	GTTAACCAAGCGCAATGATACTCCTCTCC	743–771
F034	Forward PCR primer for fragment B	CTTCCGTCATGTTGCAGGGCTTTGTCGTTA	549–578
F035	RT and reverse PCR primer for fragment B	ACTGTTTGTTTTGGCCCATGCATTATAGGATTCT	9649–9682
F029	Forward PCR primer for fragment C	GGGGAAATGTATGGCGGTTATGAAGAT	9503–9529
F030	RT and reverse PCR primer for fragment C	ACCTGGCGCTGTTTTTACGAAGTC	12,979–13,002
F002	Forward PCR primer for fragment D	CTTAAAGATTCAGGTGCGGTTGC	12,586–12,608
F003	RT and reverse PCR primer for fragment D	AGCTTGGATATGGTGTTGTACTTCTCTT	15,848–15,875
F012	Forward PCR primer for fragment E	CGCCATATTGAAAGAGGTCGTC	15,570–15,591
F016	RT and reverse PCR primer for fragment E	AAGTCCTTTCACAGCGTTATTAGA	18,877–18,900
F025	Forward PCR primer for fragment F	CGGCGAGTACGTTGAACAGATTGAC	18,996–19,020
F026	RT and reverse PCR primer for fragment F	GTATAAGTTTGCACAGTTGTTGGATTTG	22,771–22,798
F021	Forward PCR primer for fragment G	TGGCTGGCCTTTACTACACATC	22,561–22,582
F022	RT and reverse PCR primer for fragment G	ACACATACCAAGGCCATTTTACAT	24,598–24,621
C032-F	Forward PCR primer for fragment H	ATGGATTTAATACTATGGCCTCAGCACT	23,719–23,746
C032-R	RT and reverse PCR primer for fragment H	CTACCCAACGCATTAACACAAAGAA	26,580–26,604
F001	Forward PCR primer for fragment I	TATGCTGAAGGGTTTAAAATGGCTGGTG	26,672–26,699
P036	Reverse PCR primer for fragment I	TGTTGGAGGGTAATGGGGTTGAA	Designed on 3′-RT primer
3′-RT	RT primer for 3′ RACE	TGTTGGAGGGTAATGGGGTTGAA-TTTTTTTTTTTTTTTTTTTTTTTNN	29,253–poly(A) tail

### Cycle sequencing

Cycle sequencing was undertaken in 10 μl reactions containing 1× BIG DYE Mix v3.1 (Applied Biosystems, UK), sequencing primer (5 pmol) and purified PCR product (100 ng). Amplification was undertaken in a GeneAmp 2400 thermocycler (Applied Biosystems, UK) using 25 cycles of 96°C for 10 s, 50°C for 5 s and 60°C for 4 min. Unincorporated dye terminators and primers were removed by ethanol precipitation and the products were then analysed by capillary electrophoresis using an ABI 310 Genetic Analyser (Applied Biosystems, UK).

### Sequence analysis

The ‘Seqman’ program in the Lasergene-6 software package (DNASTAR Inc, USA) was used for the alignment of sequence data. Comparison of predicted FIPV 79-1146 structural and non-structural proteins with those of FCoV C1Je and FCoV C1Li were undertaken with the ‘Megalign’ program using the Jotun Hein method. The putative ribosomal frameshift element, as well as putative 5′-untranslated region (5′-UTR) and 3′-untranslated region (3′-UTR) secondary structure elements, was identified visually. Protease cleavage sites within the replicase polyprotein were predicted by alignment with the replicase polyproteins of FIPV 79-1146. The Simplot v3.5.1 program (http://sray.med.som.jhmi.edu/SCRoftware/Simplot) was used to analyse for ‘identity’ in the aligned S genes and flanking regions using a window size of 200 nucleotides and a step size of 20 nucleotides. The ‘identity’ values were calculated using a maximum likelihood probability.

## Results

### Genomic sequence of FCoV C1Je

Genomic sequence data were generated for viral RNA derived from the jejunum of a cat with a histopathologically confirmed diagnosis of FIP. The viral RNA was designated FCoV C1Je and the genomic sequence was derived from nine overlapping PCR products (seven RT-PCR and two RACE-PCR) (Fig 1). The genomic RNA sequence of FCoV C1Je comprises 29,255 nucleotides, excluding the 3′ poly(A) tail. The genomic organisation is similar to that of FIPV 79-1146 and the overall nucleotide composition is A, 28.94%; G, 21.11%; U, 32.69% and C, 17.26%. The sequence has been deposited with the GenBank database (accession number DQ848678).

### 5′-UTR and 3′-UTR

**Fig 2:**
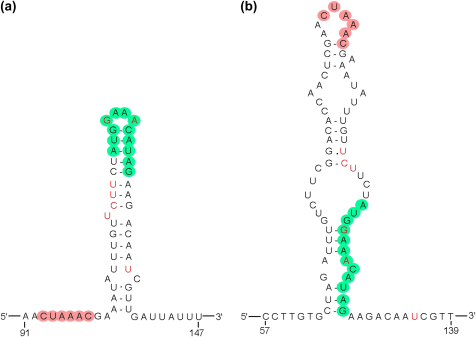
Two mutually exclusive stem loop structures (a) and (b) are predicted. The leader TAS element is highlighted in pink and a four codon ‘mini ORF’ is highlighted in green. Nucleotides that are different from those in the analogous FIPV 79-1146 structures are highlighted in red.

**Fig 3:**
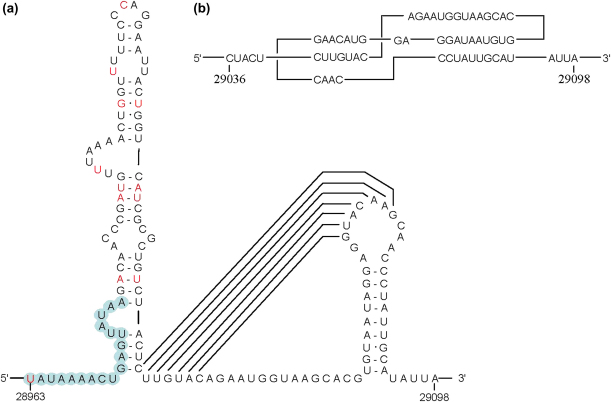
A putative molecular switch is present within the 3′-UTR region of the FCoV C1Je genomic RNA consisting of a double stem loop structure and a pseudoknot. The nucleotide positions in the FCoV C1Je genome are indicated and nucleotides differing from those in the analogous structures of FIPV 79-1146 are shown in red. The 3′ end of ORF7b is highlighted in blue.

The FCoV C1Je 5′-UTR comprises 310 nucleotides, one nucleotide less than that of FIPV 79-1146. Within this region, two putative secondary structures, the so-called ‘leader transcription-associated-sequence hairpin (LTH)' (nts 91–147) and a second stem loop structure (nts 57–139) can be predicted (Fig 2), bearing >90% nucleotide identity with similar structures found in the FIPV 79-1146 genomic RNA. As in FIPV 79-1146, these structures encompass a ‘mini-open reading frame (ORF)’ of four codons (nts 116–127) and the leader ‘transcription-associated-sequence’ (TAS) (nts 93–98), 5′-CUAAAC-3′, which is also located adjacent and upstream of six putative ORFs in the genomic RNA. The 3′-UTR of FCoV C1Je contains a putative bulged stem loop and pseudoknot, again bearing >90% nucleotide identity with the analogous structures of FIPV 79-1146 (Fig 3). As in FIPV 79-1146, these structures extend into the upstream ORF7b, which in this isolate, unlike in 79-1146, appears to be intact (see below).

### ORFs and expression products

As expected, analysis of the FCoV C1Je genomic RNA sequence data reveals the presence of six ORFs that, by comparison with other coronaviruses, can be deduced to encode the non-structural and structural proteins of the virus (Siddell et al, 2005, Gorbalenya et al, 2006). ORF1a (nts 311–12,391) and ORF1b (nts 311–20,390) encode the non-structural proteins (nsps). As in FIPV 79-1146, these ORFs overlap by 46 nucleotides and a typical coronavirus ‘slip-site’, 5′-UUUAAAC-3′ (nts 12,355–12,361), is located within the overlap. Adjacent and downstream of the ‘slip-site’ is a putative pseudoknot structure that shares 100% nucleotide identity with the putative FIPV 79-1146 pseudoknot. Amino acid comparisons of the FCoV C1Je nsps with those of FIPV 79-1146 (Table 2) reveal fairly high conservation (>90%).

**Table 2:** Comparison of predicted FCoV C1Je replicase cleavage products with those of FIPV 79-1146

Cleavage product	Polyprotein	Position in polyprotein (amino acid residues)	Size (aa)	Expression	Amino acid identity (%) with FIPV 79-1146	Putative function
nsp1	pp1a/pp1ab	1Met-Gly110	110	TI + PL^pro^	91.8	
nsp2	pp1a/pp1ab	111Val-Gly879	769	PL^pro^	90.5	
nsp3	pp1a/pp1ab	880Gly-Gly2397	1518	PL^pro^	86.9	PL^pro^(s), ADRP
nsp4	pp1a/pp1ab	2398Ser-Gln2887	490	PL^pro^ + 3CL^pro^	93.5	
nsp5	pp1a/pp1ab	2888Ser-Gln3189	302	3CL^pro^	96.4	3CL^pro^
nsp6	pp1a/pp1ab	3190Ser-Gln3483	294	3CL^pro^	90.5	
nsp7	pp1a/pp1ab	3484Ser-Gln3566	83	3CL^pro^	94.0	
nsp8	pp1a/pp1ab	3567Ser-Gln3761	195	3CL^pro^	96.9	
nsp9	pp1a/pp1ab	3762Asn-Gln3872	111	3CL^pro^	95.5	ssRNA binding
nsp10	pp1a/pp1ab	3873Ala-Gln4007	135	3CL^pro^	97.0	
nsp11	pp1a	4008Gly-Asp4026	19	3CL^pro^ + TT	94.7	
nsp12	pp1ab	4008Gly-Gln4937	929	RFS + 3CL^pro^	96.8	RdRp
nsp13	pp1ab	4938Ala-Gln5536	599	RFS + 3CL^pro^	94.7	Helicase
nsp14	pp1ab	5537Ser-Gln6055	519	RFS + 3CL^pro^	95.2	Exonuclease
nsp15	pp1ab	6056Ser-Gln6394	339	RFS + 3CL^pro^	95.9	Endoribonuclease
nsp16	pp1ab	6395Ser-Pro6694	300	RFS + 3CL^pro^ + TT	93.0	2′-*O*-Methyltransferase

nsp = non-structural protein, TI = translation initiation, TT = translation termination, RFS = ribosomal frameshift, PL^pro^ = papain-like proteinase, 3CL^pro^ = 3C-like proteinase, ADRP = ADP-ribose 1′ phosphatase, RdRp = RNA-dependent RNA polymerase, ssRNA = single stranded RNA.

**Fig 4:**
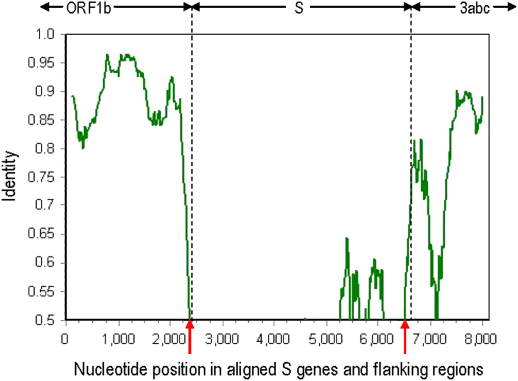
The identity of the aligned FIPV 79-1146 and FCoV C1Je S genes and flanking regions calculated using the ‘Simplot’ program is shown. An identity of 1.0 indicates regions sharing 100% nucleotide similarity. The positions of ORF1b, ORF S and ORF3abc are shown and putative sites for recombination of the FIPV 79-1146 S gene with that of CCoV are indicated with red arrows. The identity calculation was undertaken using a window size of 200 nucleotides and a step size of 20 nucleotides.

The ORFs encoding the structural proteins are ORF S (nts 20,388–24,791), ORF E (nts 25,846–26,100), ORF M (nts 26,111–26,900) and ORF N (nts 26,915–28,045) and their predicted translation products are the spike glycoprotein (S), the envelope protein (E), the membrane protein (M) and the nucleocapsid protein (N). Comparative sequence analysis shows that most of the structural proteins of FCoV C1Je are very closely related to those of FIPV 79-1146 with amino acid identities exceeding 90% (Table 3). However, this is not the case for the S protein, which shares only 43.3% amino acid identity between the two isolates. Since 80–90% of field strain FCoVs are serotype I (Hohdatsu et al, 1992, Benetka et al, 2004), the FCoV C1Je isolate is likely to be a type I virus. The amino acid identity of the FCoV C1Je S protein with that of published FCoV type I S protein sequence data is very high (>85%) (Table 4) confirming that it as a type I strain. FIPV 79-1146 is a serotype II isolate and this is illustrated by the finding that the FIPV 79-1146 S protein shares strong (>90%) amino acid identity with published CCoV isolates (Table 5). Previous predictions based on limited sequence data have identified type II FCoV strains with a double recombination event occurring in the 5′ half of ORF1b (Herrewegh et al 1998) and between the S and M genes (Motokawa et al 1996). The FIPV 79-1146 and FCoV C1Je S genes and their flanking sequences were aligned and a graph of the relative nucleotide identities was plotted. This was used to make a prediction for the putative crossover sites of FIPV 79-1146 with CCoV (Fig 4). The upstream crossover site was predicted to lie at position 20,119–20,200 (3′ end of ORF1b) and the downstream crossover site at position 24,328–24,329 (3′ end of S gene) in the FIPV 79-1146 genome (GenBank accession number DQ010921). A short conserved region of approximately 900 nucleotides lying within the predicted recombination site is likely to represent the heptad repeat sequences in the S2 subdomain which are essential for fusogenic activity and are conserved throughout coronaviruses (Bosch et al, 2003, de Haan and Rottier, 2005).

**Table 3:** Comparison of predicted FCoV C1Je non-structural, structural and accessory proteins with those of FIPV 79-1146 and FCoV C1Li

ORF (nucleotide positions)	Translation product (amino acids)	Amino acid identity (%)	
		FIPV 79-1146	FCoV C1Li
ORF1a (311–12,391)	Polyprotein 1a (4026)	90.8	–
ORF1ab (312–20,390)	Polyprotein 1ab (6692)	92.7	–
ORF S (20,388–24,791)	Spike glycoprotein (1466)	43.3	100
ORF3a (24,803–25,015)	Accessory protein 3a (69)	67.1	100
ORF3b (24,963–25,175)	Accessory protein 3b (69)	50.0	100
ORF E (25,846–26,100)	Small membrane protein (81)	91.5	100
ORF M (26,111–26,902)	Membrane protein (262)	94.3	100
ORF N (26,915–28,045)	Nucleocapsid protein (376)	91.5	100
ORF7a (28,050–28,355)	Accessory protein 7a (100)	94.1	100
ORF7b (28,360–28,980)	Accessory protein 7b (206)	91.7	100

**Table 4:** Direct amino acid comparison of the FCoV C1Je S protein with the S proteins of published FCoV type I isolates

Type I FCoV strain	GenBank database ID	S protein amino acid identity (%)
Black	AB088223	87.1
Ku-2	AAB47503	86.7
UCD-1	AB088222	87.8
NTU2/R	AAZ86077	89.3

**Table 5:** Direct amino acid comparison of the FIPV 79-1146 S protein with the S proteins of published CCoV and FCoV type II isolates

Viral strain	GenBank database ID	S protein amino acid identity (%)
CCoV CCV-6	A22882	91.2
CCoV K378	X77047	91.8
FCoV 79-1146	X06170	99.8
FCoV 79-1683	X80799	95.1

**Fig 5:**
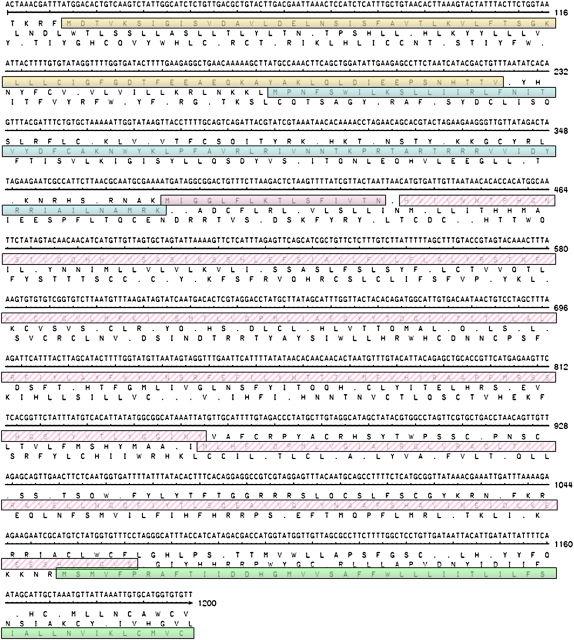
The gene 3 region of FCoV C1Je is shown. ORF3a is highlighted in yellow, ORF3b in turquoise and ORF3c, which terminates prematurely, is shown in pink. Extension of the degenerate ORF3c to overlap with ORF E (green) would require only two nucleotide mutations (one change and one deletion) and is illustrated in pale pink.

Analysis of the accessory gene 3 cluster of the FCoV C1Je genome reveals ORFs corresponding to 3a and 3b as found in the FIPV 79-1146 isolate. Although the encoded proteins appear to be analogous to those of FIPV 79-1146 and are of similar length, the amino acid conservation is not high (Table 3). This may reflect the fact that these ORFs are believed to be dispensable for replication in cell culture and are thus unlikely to be conserved in the laboratory-adapted strain of FIPV 79-1146 (Haijema et al 2004). As in FIPV 79-1146, ORF3c of FCoV C1Je appears to be defunct and has a stop codon after only 16 amino acids. However, the introduction of only two nucleotide mutations would enable extension of the reading frame to overlap with that of ORF E (Fig 5). Analysis of the accessory gene 7 region of the FCoV C1Je genome identifies two ORFs, which have translation products sharing high amino acid identity with proteins 7a and 7b of FIPV 79-1146. However, unlike the FIPV 79-1146 ORF7b, which appears to terminate early as a result of a single nucleotide mutation (C_28,374_ to U_28,374_) (Dye and Siddell 2005), the FCoV C1Je ORF7b is intact.

### Comparison of enteric and non-enteric FCoVs

Using the strategy outlined earlier, the sequence of the 3′-proximal 10 kb (nts 19,060–29,255) of viral RNA derived from liver tissue of the same cat was determined. This sequence was designated FCoV C1Li. When the nucleotide and amino acid sequences of FCoV C1Je (enteric) and FCoV C1Li (non-enteric) were compared, the sequences were found to be identical. Thus, *cis*-acting RNA elements in this region, as well as the structural and accessory genes and encoded proteins of the two RNAs, were found to share 100% identity (Table 3).

## Discussion

The genomic RNA sequence of FCoV C1Je provides the first full-length sequence of a field strain FCoV. Comparisons with the genomic RNA of FIPV 79-1146 show that as expected it has a very similar genome organisation and predictions made for the ORFs and *cis*-acting elements within FIPV 79-1146 are equally valid for FCoV C1Je.

However, more detailed comparative sequence analysis of the field strain type I RNA (FCoV C1Je) with that of the type II laboratory strain (FIPV 79-1146) does reveal a number of interesting differences. As expected there was significant diversity in the S gene region resulting from the evolutionary recombination of FIPV 79-1146 with CCoV (Herrewegh et al 1998). The discontinuous transcription method used by coronaviruses is very similar to that of the template switching that occurs during similarity-assisted or high frequency copy-choice RNA recombination (Sawicki and Sawicki, 2005, Sawicki et al, 2006). Recombination is a common phenomenon amongst coronaviruses (Wang et al, 1993, Schaad and Baric, 1994, Collisson et al, 1995, Rest and Mindell, 2003, Johnson et al, 2005) and it may have greater evolutionary significance than the slow process of genetic drift. It provides a mechanism for the rapid formation of new viral strains with dramatically altered tropisms and pathogenicity, which can have a significant impact on host disease. First, it raises the threat of potentially lethal phenotypes emerging over a very short evolutionary time scale and second, it compounds the difficulties of vaccine production resulting from the heterogeneity of virus populations. This again emphasises the importance of investigating current field strain viruses rather than relying solely on laboratory-adapted isolates.

Second, comparisons of the replicase proteins of FIPV 79-1146 and FCoV C1Je showed them to be extremely similar and 15 of the 16 non-structural proteins had amino acid identities of >90%. The amino acid identity of the nsp3 regions was, however, slightly lower at 86.9% and this was largely due to the presence of an extra 65 amino acids in FCoV C1Je. The closely related coronavirus Transmissible Gastroenteritis Virus (TGEV) also contains similar additional amino acids within its nsp3 region (Penzes et al 2001) and it is most likely that a 65 amino acid deletion has occurred in FIPV 79-1146 during its passage in cell culture. The presence of this deletion in FIPV 79-1146 suggests that this particular region is not required for viral replication, at least in vitro.

Analysis of the accessory gene regions of the clinical strain is particularly interesting. It has long been suspected that these genes confer a selective advantage in vivo but are not required in vitro (Herrewegh and Vennema, 1995, Kennedy et al, 2001, Haijema et al, 2003), and that analysis of sequences derived directly from clinical material might, therefore, provide some insight into their importance. Both gene 3 and gene 7 regions have also been implicated in viral pathogenicity. For example, mutant viruses containing either 3abc or 7ab cluster deletions multiply well in cell culture but show an attenuated phenotype in the cat (Haijema et al 2004). Interestingly, cats vaccinated with either of these viral mutants showed some protection against a lethal homologous challenge but vaccination with a mutant virus lacking both gene clusters provided no protection (Haijema et al 2004). It is certainly noteworthy that ORF7b is intact in the clinical strain but not in FIPV 79-1146. This region, along with ORF3c, has been previously implicated in viral pathogenicity. For example, investigation of paired FECV/FIPV samples from various geographical locations has suggested that the FIPV may arise from deletion mutations in the FECV accessory genes (Vennema et al 1998). Further investigation into the function of the encoded 7b protein would certainly be rewarding. It has previously been shown that the 3c gene region of FIPV 79-1146 is degenerate but that it could be restored by just two nucleotide insertions that would enable its extension to overlap with ORF E (Dye and Siddell 2005). It was, therefore, suspected that the ORF3c of FCoV C1Je might be intact, but this was not the case. It is difficult to speculate on the implications of this finding but is does at least suggest that the 3c protein may have restricted functional significance in vivo.

In this paper, viral RNA extracted from two different tissue samples, one enteric (jejunum) and one non-enteric (liver), was sequenced and compared in an attempt to investigate the possibility of genetic differences that might account for the enhanced pathogenicity of FIPV compared with FECV. The finding of a 100% nucleotide identity in the structural and accessory gene regions of the enteric virus (FCoV C1Je) and the non-enteric virus (FCoV C1Li) does not support this view. In fact, it provides a powerful argument opposing the ‘internal mutation theory’. However, there are some limitations that may also explain this result. For example, consensus sequencing will mask minority virus populations and it is possible that a second viral isolate is present at low levels within the cat 1 jejunum sample. One potential model would be that following immune impairment in cats with well-progressed FIP disease, pathogenic FIPV is able to replicate uncontrollably and migrates back to the gut where viral loads are able to exceed those of the enteric FECV strains. Secondly, it is possible that the important determinants of pathogenicity are located in the proteins encoded in the replicase gene region. It is certainly conceivable that interaction between proteins of the replicase complex and cellular proteins could have critical importance and that alterations in this interaction could lead to altered pathogenicity. It is known, for example, that significant CD4 and CD8 T cell epitopes are located in the replicase proteins of Murine Hepatitis Virus (MHV) (Stohlman et al 1993) and it is already clear that some coronavirus non-structural proteins have profound effects on cellular processes such as deubiquitination and Adenosine Diphosphate (ADP)-ribose metabolism (Barretto et al, 2005, Putics et al, 2005). The next step would certainly be to obtain the full genomic RNA sequence of FCoV C1Li so that the replicase gene regions can be compared.

There are still many unanswered questions regarding the molecular epidemiology of FCoV. For example, does the FCoV strain within a defined multi-cat population remain conserved over time or are new strains constantly emerging? If new strains do emerge, are they the result of original strain mutation or ‘de novo’ infections from external sources? Are all coronavirus positive cats within a household infected with the same viral strain and are some cats co-infected with several FCoV strains simultaneously? Is the FCoV strain present in the gut always a reflection of the predominant systemic strain, and similarly, do cats with FIP excrete pathogenic FIPV or only FECV? Is there a consistent mutation associated with pathogenicity? Various authors have previously addressed many of these questions (Addie, 2000, Addie and Jarrett, 2001, Pedersen, 2002). However, only small sections of the viral genome have so far been considered and interpretation of the findings has proved difficult. Now that the genomic RNA sequence of a type I clinical strain has been elucidated it will be much easier and quicker to sequence more extensive specific regions from further viral field strains. It is hoped that this will help to shed more light on the findings of future epidemiological studies and increase their significance.

Lastly, the genomic RNA sequence of FCoV C1Je provides a solid foundation for the construction of a full-length cDNA copy of a field strain FCoV. This could be used as the basis for production of a reverse genetics system, a tool that would enable us to study FCoV molecular biology and pathogenesis. Studying a clinical isolate in such a way is likely to provide more clinically relevant information than studying FIPV 79-1146 and would have greater potential for use in any future attempt to make a live attenuated vaccine.
